# Association between under-dose of enzyme replacement therapy and quality of life in adults with late-onset Pompe disease in China: A retrospective matched cohort study

**DOI:** 10.1371/journal.pone.0310534

**Published:** 2024-09-17

**Authors:** Huanyu Zhang, Shanquan Chen, Richard Huan Xu, Siyue Yu, Jiazhou Yu, Dong Dong

**Affiliations:** 1 Shenzhen Research Institute, The Chinese University of Hong Kong, Shenzhen, China; 2 Clinical Big Data Research Center, The Seventh Affiliated Hospital, Sun Yat-sen University, Shenzhen, China; 3 International Centre for Evidence in Disability, London School of Hygiene & Tropical Medicine, London, United Kingdom; 4 Department of Rehabilitation Sciences, The Hong Kong Polytechnic University, Hong Kong SAR, China; 5 Faculty of Medicine, Jockey Club School of Public Health and Primary Care, The Chinese University of Hong Kong, Hong Kong SAR, China; Kwame Nkrumah University of Science and Technology, GHANA

## Abstract

**Background:**

Due to the high cost of enzyme replacement therapy (ERT), most of adults with late-onset Pompe disease (LOPD) who received ERT used the medication with insufficient dosefs in China.

**Objective:**

To compare the change in quality of life (QoL) between adults with LOPD receiving under-dose ERT and no ERT, and identify factors associated with the change of QoL.

**Methods:**

A retrospective matched cohort study was conducted among adult patients with LOPD in a nationwide Pompe registry in China. Eligible participants were those who completed two investigations, and didn’t expose to ERT at baseline or before. The treated group were those who used ERT during follow-up; the untreated group received general care. The treated and untreated group were matched with a ratio of 1:2. QoL was assessed by the SF-12 and EQ-5D-5L. The dose of ERT was evaluated by the ratio of actual vials patients used divided by the indicated vials patients should use. The treated patients were further classified into mild and severe under-dose users by the median ratio. Multivariate linear regression analyses were performed to estimate the average treatment effect in the treated groups and identify factors associated with the changes of QoL scores.

**Results:**

The study sample included 5 mild under-dose users, 6 severe under-dose users, and 22 untreated participants. Compared with the untreated group, mild under-dose ERT had no significant effect on the changes of QoL scores. In contrast, severe under-dose ERT was associated with a decline of physical QoL (β = -6.19, *p* = 0.001), but an increase of overall health state (β = 19.69, *p* = 0.032). A higher score of physical QoL (β = -0.74, *p* = 0.001) and overall health state (β = -0.69, *p*<0.001) at baseline was associated with decline in corresponding scores at follow-up. Being female was a contributor to the worsening of the overall health state (β = -22.79, *p* = 0.002), while being employed or at school was a predictor of improvement in mental QoL (β = 5.83, *p* = 0.002).

**Conclusions:**

A Pompe-disease specific instrument based on patient experiences is warranted to closely monitor changes in QoL on a routine basis. It is desirable for patients with severe under-dose ERT to discuss with physicians whether to adjust treatment strategies.

## Introduction

Pompe disease, also known as glycogen storage disease type Ⅱ, is an inherited metabolic disorder in which a deficiency of the lysosomal enzyme acid α-glucosidase (GAA) leads to accumulations of glycogen in various tissues. All muscle groups of patients can be affected, especially skeletal muscles and respiratory system [1], increasing the risk of wheelchair or ventilator dependency [2]. Late-onset Pompe disease can occur at any time in children and adults with a wide spectrum of symptoms and complications [3–5], leading to a decline in quality of life [2], limited ability to work or study [6], disabilities or even death [3,7].

Although Pompe disease is a rare condition, it currently has specific drug treatment, i.e., enzyme replacement therapy (ERT) with alglucosidase alfa. Long-term use of ERT could improve or stabilize ambulation and respiratory function, and reduce mortality among patients with LOPD [1]. Similar to other orphan drugs for rare diseases, ERT to treat Pompe disease is very expensive. It is estimated that the average annual cost of ERT for an adult with LOPD is approximately RMB 3,000,000 (USD 428,500), which is over four times of the average annual household income in China [8]. Given the low reimbursement rate of social and commercial insurance for ERT [8], a heavy financial burden still exists for patients with Pompe disease in China, especially for adult patients who usually need higher doses of ERT determined by body weight in comparison with pediatric patients.

Cost-related medication under-dose use is a common clinical practice that occurs in multiple chronic diseases due to financial hardship and inadequate prescription coverage [9,10]. Compared with standard-dose use of medications, under-dose use could compromise the efficacy of treatment, and thus increase the risk of disease-related complications, reduced quality of life, and mortality [11–13]. In many developed countries, ERT for Pompe disease is fully reimbursed despite its high cost. However, in developing countries like China, insufficient reimbursement is common [8,14]. ERT is the only specific pharmacotherapy to treat Pompe disease without any alternative so far. Under these circumstances, Chinese adults with LOPD who are in great needs of ERT would discuss with their physicians and choose to use a reduced dose. By doing this, they could afford it or make their medication supply last longer, just like other chronically ill patients who underuse prescription medications because of cost concerns [9,15]. In this patient-physician communication, under-dose use of ERT is a therapeutic decision under nonideal conditions that physicians need to take account of the best interests of their patients not only from a clinical perspective, but also from financial, emotional, and psychosocial perspectives [16].

Since few adult patients with LOPD used ERT with a standard dose in China, it is important to assess whether under-dose use of ERT could improve or stabilize health outcomes from a patient perspective compared with receiving no ERT. Under this circumstance, quality of life (QoL) is a suitable outcome measure that provides a multidimensional perspective of an individual’s subjective perception on health [17]. Several previous studies have focused on the effect of ERT on QoL among patients with LOPD [18,19]. Nonetheless, to our best knowledge, no studies have been conducted to examine the association between under-dose ERT and QoL among adult patients with LOPD. The primary objective of this study is to compare the change of QoL between adults with LOPD receiving under-dose ERT and no ERT. The secondary objective is to identify factors associated with the change of QoL.

## Methods

### Study design and sample

A retrospective matched cohort study was conducted in adults aged 18 years or older who had a definitive diagnosis of LOPD and registered in the China Pompe Care Center (CPCC), the only national Pompe patient organization in China. The patient registry conducted a survey every 12 months on a routine basis. The baseline data were collected in December 1, 2019. An extension of 2 months was implemented in the second routine survey due to the COVID-19 crisis by the end of 2020. Hence, the follow-up data were collected after 14 months in February 1, 2021.

**Fig 1:**
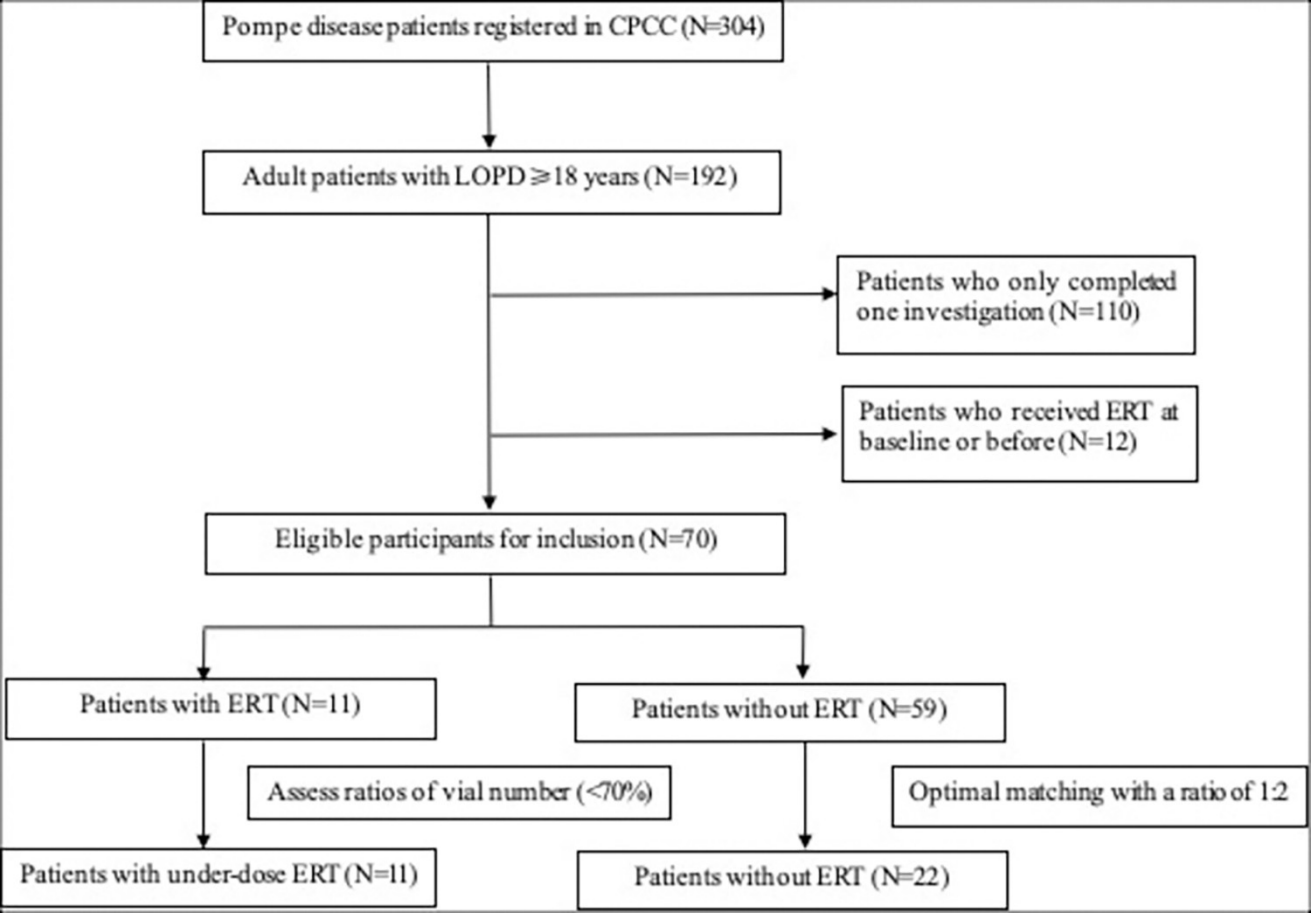
Flowchart of the study sample for analyses.

Eligible participants were those who completed two investigations and didn’t expose to ERT at baseline or before. The treated group were those who used ERT during follow-up. Propensity scoring method was employed to construct the untreated group from the remaining participants who received general care, with propensity scores computed via logistic regression. The optimal matching was conducted between the treated and untreated patients with a ratio of 1:2 based on age, sex, level of dependence on assistive devices (none, some, or a lot), and geographic location (rural or urban). The level of dependence on assistive devices could represent patients’ physical abilities, and it is an important predictor of physical QoL for patients with Pompe disease [8]. Furthermore, since healthcare resources are unevenly distributed depending on the geographic location in China, patients in urban areas have more access to high-quality healthcare compared with patients residing in rural areas [20]. Hence, the aforementioned variables were adopted for matching to control for the potential imbalance between the treated and untreated group. We employed the MatchIt package in the R software to calculate propensity scores and construct a new sample composed of the treated and untreated group that has the closest propensity score. After propensity score matching, the standardized mean differences were smaller than 0.1 for all covariates, a threshold commonly indicated as sufficient balance [21]. The procedure of constructing the study sample was summarized in **Fig 1**. The characteristics of the participants before matching are presented in **S1 Table**.

A self-administered online survey was employed to collect the data. Before the survey was formally distributed, we invited social scientists from the field of quality of life research and neurologists specialized in Pompe disease to review the contents and structure of the questionnaire. Meanwhile, a pilot study was conducted in a small sample of patients and caregivers from the patient organization to examine if they could understand each question. When the survey was officially conducted, participants’ responses were uploaded to an online platform and manually checked by two researchers. Missing values as well as answers with logic errors were verified by trained researchers by confirming with the patient or caregiver.

This study has been approved by the Survey and Behavioural Research Ethics Committee of the Chinese University of Hong Kong (No.: SBRE-18-268). All the participants were required to read the consent form and click the “consent to participate” button before entering the study. All the individual participants were de-identified during or after data collection.

### Data collection

#### Quality of life

Since there is no Pompe disease-specific tool to assess QoL so far, we used two validated Chinese versions of generic instruments to assess QoL at both baseline and follow-up, including the 12-item Short-Form Health Survey (SF-12) and EuroQol 5-dimension instrument with five-level scale (EQ-5D-5L). The SF-12 is more sensitive to measure differences associated with less severe morbidity, while EQ-5D is more useful for measuring health in people with more morbidity [22,23]. The combination of the EQ-5D and SF-12 could increase the breadth of health domains measured for various study objectives [23].

Two component scores from the SF-12 were calculated, including physical component score (PCS) represented by 2-item physical function, 2-item role physical, 1-item bodily pain, and 1-item general health; and mental component score (MCS) represented by 1-item vitality, 1-item social function, 2-item role emotion, and 2-item mental health [24,25]. The component scores are transformed to a standardized 0–100 scale, with higher scores indicating better health functioning.

EQ-5D-5L comprises a descriptive system and a visual analogue scale (VAS). The descriptive system consists of five dimensions: mobility, self-care, usual activities, pain/discomfort, and anxiety/depression [26]. The VAS measures the participant’s perception of their overall health on a scale of 0 to 100. By linking the descriptive system to a value set, an index value can be derived based on the general population’s preferences in a specific country. Previous studies have shown a ceiling effect in EQ-5D dimension scores, but using the VAS and index value helps mitigate this limitation [22,27]. Therefore, we employed the VAS and index score of the EQ-5D-5L to assess changes in the participants’ QoL. The index score for each participant was computed according to the preferences of Chinese population in this study [28].

#### Assessment of ERT use

Patients who received ERT were further investigated the actual doses they had used during follow-up. The approved dosage regimen of Myozyme® in China is 20 mg/kg of body weight administered twice every month. Alglucosidase alfa 50 mg powder is contained in one vial for reconstituted solution for infusion. The participants were required to report their body weight and the actual number of vials they had used during follow-up. The dose of ERT use was evaluated by the ratio of actual vials patients used divided by the indicated vials patients should use. We found that all the participants with ERT used the medication with a ratio of vial number less than 70%. Although there is no consensus on a threshold for measuring patients’ adherence to pharmacotherapy, most of the studies used the threshold of 80% to indicate a good adherence [29]. Hence, we considered all the participants with ERT as under-dose ERT users in this study. To identify a dose-effect relationship between the level of dose and QoL, we further divided patients with ERT into two groups by the median ratio of vial number. Patients who used ERT above the median ratio were classified as mild under-dose ERT users, otherwise they were classified as severe under-dose ERT users. Hence, 5 participants were classified as mild under-dose users, while the rest of 6 participants were considered as severe under-dose users (**S2 Table**).

#### Demographic and other baseline characteristics

The following demographic characteristics of the participants were collected at baseline, including age, sex (male or female), geographic location, being employed or at school (yes or no), and education (middle school or lower, high school, or above high school).

Considering the potential factors associated with QoL in the context of Pompe disease [8,20,30,31], we assessed other baseline characteristics including disease severity, measured by wheelchair use (yes or no), ventilator use (yes or no), and level of dependence on assistive devices (none, some, or a lot), and perceived social support measured by the Chinese version of 19-item Medical Outcomes Study Social Support Survey (MOS-SSS-CM) in this study [32]. Four subscale scores of social support, including tangible support, emotional/ informational support, positive social interactions, and affectionate support, are transformed to a standardized 0–100 scale, with a higher score indicating better perceived support [32].

#### Socioeconomic characteristics

We also investigated the economic burden on participants and their families, measured by catastrophic health expenditure (CHE) in this study. Participants were asked to report the annual out-of-pocket (OOP) health expenditures and family incomes in the year of 2019 and 2020 at baseline and follow-up, respectively; average OOP health expenditures and family incomes in these two years were calculated for each participant. CHE (yes or no) was judged by analyzing whether OOP health expenditure exceeded 10% of family income. Furthermore, participants were investigated at follow-up whether they participated in any rare disease-specific insurance or medical assistance program supported by the local government, foundation, patient organization, or private insurance company.

### Statistical analyses

The characteristics of the participants were described between the treated and untreated group. Continuous variables were reported as mean (standard deviations [SD]) and compared by Wilcoxon test. Categorical variables were reported as frequencies (percentages) and compared by Fisher’s Exact test. We used Kruskal-Wallis test to compare the differences in four indicators of QoL scores at baseline and follow-up, respectively, between the group with mild under-dose, severe under-dose ERT, and without ERT. Paired sample Wilcoxon test was also adopted to test the mean changes of QoL scores between baseline and follow-up among the three groups.

We used the changes of QoL scores between baseline and follow-up as key outcomes. Univariate analyses were performed including the Mann-Whitney U-test for binary variables, Kruskal-Wallis test for categorical variables with more than two categories, and Pearson correlation test for continuous variables. We further conducted multivariate linear regression analyses to estimate the average treatment effect in the treated groups and identify factors associated with the changes of QoL scores. Variables with *p*<0.1 in univariate analyses and QoL scores at baseline were further included in the multivariate linear regression models.

All statistical analyses were conducted using the R software (version 4.2.2). Statistical significance was defined as *p*<0.05.

## Results

In total, 304 Pompe disease patients registered in CPCC, 192 of whom were adult patients with LOPD. Among 82 participants who completed two investigations at baseline and follow-up, 70 were not using ERT at baseline or before. When assessing ERT status during follow-up, all the 11 participants who chose to use the medication were considered as under-dose ERT users, while 59 participants were not using ERT. After performing the optimal matching, 11 treated and 22 untreated participants were included as the study sample (**Fig 1**).

In **Table 1**, of the 33 participants included, 15 (45.5%) were females, 21 (63.6%) located in rural areas, and 24 (72.7%) were not employed or at school, with a mean age of 30.2 (7.2). Compared with the sample before matching (**S1 Table**), the sample after matching presented a relatively higher level of disease severity, with the majority (81.8%) of the study sample depending on assistive devices a lot, 21.2% using the wheelchair, and 93.9% using the ventilator. Most (81.8%) of the participants with under-dose ERT suffered from CHE, while this proportion was 40.9% in patients without ERT use (*p* = 0.034).

**Table 1:** Demographic and socioeconomic characteristics of the study sample.

	Total^a^ N = 33	Not ERT^a^ N = 22	Under-dose ERT^a^ N = 11	*p-*value^b^
**Age**	30.2(7.2)	30.0(7.4)	29.9(7.1)	0.775
**Sex**				
Female	15(45.5%)	10(45.5%)	5(45.5%)	1.000
Male	18(54.5%)	12(54.5%)	6(54.5%)	
**Reliance on devices**				1.000
Some	6(18.2%)	4(18.2%)	2(18.2%)	
A lot	27(81.8%)	18(81.8%)	9(81.8%)	
**Geographic location**				1.000
Rural	21(63.6%)	14(63.6%)	7(63.6%)	
Urban	12(36.4%)	8(36.4%)	4(36.4%)	
**Wheelchair use**				0.661
No	26(78.8%)	18(81.8%)	8(72.7%)	
Yes	7(21.2%)	4(18.2%)	3(27.3%)	
**Ventilator use**				1.000
No	2(6.1%)	1(4.5%)	1(9.1%)	
Yes	31(93.9%)	21(95.5%)	10(90.9%)	
**Employment/school**				0.438
No	24(72.7%)	17(77.3%)	7(63.6%)	
Yes	9(27.3%)	5(22.7%)	4(36.4%)	
**Education**				0.587
Middle school or lower	10(30.3%)	8(36.4%)	2(18.2%)	
High school	12(36.4%)	7(31.8%)	5(45.5%)	
Above high school	11(33.3%)	7(31.8%)	4(36.4%)	
**Social support**				
Tangible support	67.2(24.8)	66.8(27.0)	68.2(20.6)	1.000
Emotional/informational support	47.3(23.5)	48.0(23.7)	46.0(24.3)	0.924
Positive social interactions	47.9(24.4)	47.2(25.6)	49.4(23.0)	0.631
Affectionate support	46.7(27.2)	45.1(27.8)	50.0(26.9)	0.440
**Any rare disease-specific insurance during follow-up**				0.065
No	20(60.6%)	16(72.7%)	4(36.4%)	
Yes	13(39.4%)	6(27.3%)	7(63.6%)	
**Any medical assistance program during follow-up**				0.146
No	27(81.8%)	20(90.9%)	7(63.6%)	
Yes	6(18.2%)	2(9.1%)	4(36.4%)	
**Catastrophic health expenditure**				**0.034**
No	15(45.5%)	13(59.1%)	2(18.2%)	
Yes	18(54.5%)	9(40.9%)	9(81.8%)	

^a^ Data are presented as mean (SD) for continuous variables and number (percentage) for categorical variables.

^b^ Bold values indicate statistical significance which is defined as *p*-value < 0.05.

**Table 2** presents the four indicators of QoL scores at baseline and follow-up between patients with mild under-dose ERT, severe under-dose ERT, and without ERT. No significant differences were found between the three groups in four indicators of QoL scores at baseline and follow-up (*p*>0.05). **Table 3** presents the results of paired sample analyses in the three groups. A significant decline of 7.1(2.7) in SF-12 PCS was found in the group with severe under-dose ERT (*p* = 0.031).

**Table 2:** The four indicators of QoL scores at baseline and follow-up between patients with mild under-dose ERT, severe under-dose ERT, and without ERT.

	Baseline				Follow-up			
	Not ERT^a^	Mild under-dose ERT^a^	Severe under-dose ERT^a^	*p*-value	Not ERT^a^	Mild under-dose ERT^a^	Severe under-dose ERT^a^	*p*-value
SF-12 PCS	36.3(3.8)	38.2(1.2)	40.9(6.1)	0.128	37.9(4.5)	39.7(4.3)	33.8(3.9)	0.085
SF-12 MCS	37.1(7.7)	35.3(5.2)	41.3(2.7)	0.193	35.2(10.6)	33.9(7.4)	40.2(6.2)	0.283
EQ-5D index score	0.360(0.320)	0.219(0.305)	0.602(0.232)	0.157	0.352(0.381)	0.310(0.522)	0.712(0.151)	0.206
EQ-5D VAS	52.0(29.0)	29.6(28.8)	46.3(19.4)	0.222	46.3(23.7)	57.8(40.0)	67.0(21.9)	0.216

^a^ Data are presented as mean (SD).

**Table 3:** The mean changes of QoL scores between baseline and follow-up in patients with mild under-dose ERT, severe under-dose ERT, and without ERT.

	Not ERT		Mild under-dose ERT		Severe under-dose ERT	
	Mean change^a^	*p*-value	Mean change^a^	*p*-value	Mean change^a^	*p*-value^b^
SF-12 PCS	1.7(5.2)	0.224	1.5(5.4)	1.000	-7.1(2.7)	**0.031**
SF-12 MCS	-1.9(9.6)	0.509	-1.4(4.0)	0.625	-1.0(4.8)	0.844
EQ-5D index score	-0.009(0.240)	0.639	0.090(0.238)	0.438	0.110(0.182)	0.313
EQ-5D VAS	-5.7(35.4)	0.509	28.2(30.6)	0.125	20.7(27.1)	0.156

^a^ Data are presented as mean (SD).

^b^ Bold values indicate statistical significance which is defined as *p*-value < 0.05.

The results of the univariate analyses of changes in four indicators of QoL scores are presented in **Table 4**. At the level of *p*<0.1, factors that were significantly associated with the change of SF-12 PCS were ERT status (*p* = 0.003) and participation in any medical assistance program (*p* = 0.059); sex (*p* = 0.058) and being employed or at school (*p* = 0.045) were significantly associated with the change of SF-12 MCS. Sex (*p* = 0.041) was significantly associated with the change of EQ-5D index score; significant contributors to the change of EQ-5D VAS included ERT status (*p* = 0.049) and sex (*p* = 0.015).

**Table 4:** Univariate analyses of the changes of four indicators of QoL.

	Change in SF-12 PCS		Change in SF-12 MCS		Change in EQ-5D index score		Change in EQ-5D VAS	
	Value^a^	p-value^b^	Value^a^	p-value^b^	Value^a^	p-value^b^	Value^a^	p-value^b^
**ERT use**		**0.003**		0.999		0.462		**0.049**
Not use	1.65(5.17)		-1.90(9.56)		-0.009(0.24)		-5.68(35.4)	
Mild under-dose	1.50(5.43)		-1.36(3.97)		0.090(0.238)		28.2(30.6)	
Severe under-dose	-7.11(2.73)		-1.04(4.80)		0.110(0.182)		20.7(27.1)	
**Age**	0.06(-0.29~0.39)	0.752	-0.18(-0.49~0.18)	0.328	-0.013(-0.35~0.33)	0.943	-0.09(-0.42~0.27)	0.634
**Sex**		0.240		**0.058**		**0.041**		**0.015**
Female	1.07(6.58)		-3.70(6.64)		-0.049(0.241)		-11.90(30.6)	
Male	-0.83(5.17)		0.04(8.97)		0.092(0.206)		17.70(34.4)	
**Reliance on devices**		0.624		0.870		0.762		0.293
Some	0.64(4.6)		-2.72(6.83)		0.050(0.164)		-7.00(30.3)	
A lot	-0.10(6.14)		-1.43(8.46)		0.023(0.245)		6.74(36.6)	
**Geographic location**		0.896		0.779		0.562		0.454
Rural	-0.03(5.26)		-2.24(8.2)		0.010(0.255)		7.43(38.6)	
Urban	0.14(6.98)		-0.65(8.18)		0.059(0.184)		-1.33(30.2)	
**Employment/school**		0.613		**0.045**		0.952		0.321
No	-0.35(6.04)		-3.15(8.44)		0.023(0.226)		0.08(35.8)	
Yes	1.05(5.45)		2.30(5.77)		0.042(0.255)		15.30(34.2)	
**Education**		0.592		0.862		0.827		0.577
Middle school or lower	1.43(5.58)		-2.43(11.6)		0.024(0.287)		-4.30(43.6)	
High school	-0.21(5.98)		-2.17(5.8)		0.049(0.19)		14.30(30.7)	
Above high school	-0.97(6.21)		-0.40(7.06)		0.009(0.234)		1.00(32.9)	
**Social support**								
Tangible support	-0.02(-0.36~0.33)	0.931	-0.19(-0.50~0.16)	0.277	0.036(-0.31~0.37)	0.842	0.00(-0.35~0.34)	0.979
Emotional support	-0.10(-0.43~0.25)	0.580	0.16(-0.19~0.48)	0.373	0.010(-0.33~0.35)	0.955	0.07(-0.28~0.41)	0.679
Positive social interactions	-0.19(-0.50~0.16)	0.280	0.24(-0.11~0.54)	0.178	0.007(-0.34~0.35)	0.971	0.05(-0.30~0.39)	0.784
Affectionate support	-0.14(-0.46~0.22)	0.452	0.08(-0.27~0.41)	0.649	-0.006(-0.35~0.34)	0.972	0.07(-0.28~0.41)	0.691
**Any rare disease-specific insurance**		0.897		0.473		0.581		0.407
No	0.26(5.84)		-1.16(9.47)		0.012(0.197)		2.45(28.8)	
Yes	-0.31(6.05)		-2.43(5.64)		0.053(0.281)		7.00(45.1)	
**Any medical assistance program**		**0.059**		0.414		0.944		0.469
No	0.94(5.5)		-1.45(8.06)		0.030(0.211)		2.78(31.1)	
Yes	-4.06(6.03)		-2.61(9)		0.018(0.329)		10.80(54.5)	
**Catastrophic health expenditure**		0.575		0.600		0.159		0.128
No	0.38(5.84)		-1.80(6.82)		-0.029(0.173)		-5.27(26.6)	
Yes	-0.25(5.99)		-1.54(9.23)		0.076(0.264)		12.20(40.6)	

^a^ Values are presented as mean (SD) for categorical variables and correlation coefficients for continuous variables.

^b^ Bold values indicate statistical significance which is defined as *p*-value < 0.1.

**Table 5** presents results on multivariate linear regression analyses. Compared with the group without ERT, severe under-dose ERT was associated with a decline in SF-12 PCS (β = -6.19, *p* = 0.001), while it was associated with an increase in EQ-5D VAS (β = 19.69, *p* = 0.032). On the contrary, mild under-dose was not significantly associated with the changes of four indicators of QoL scores compared with the patients without ERT (*p*>0.05). Furthermore, SF-12 PCS (β = -0.74, *p* = 0.001) and EQ-5D VAS at baseline (β = -0.69, *p*<0.001) were negatively associated with SF-12 PCS and EQ-5D VAS at follow-up, respectively. Being employed or at school was associated with an increase in SF-12 MCS (β = 5.83, *p* = 0.002). Female sex was associated with a decline in EQ-5D VAS (β = -22.79, *p* = 0.002).

**Table 5:** Multivariate linear regression analyses of the changes of four indicators of QoL.

Outcomes	Variables	Estimates	*p*-value^a^
Change in SF-12 PCS	PCS at baseline	-0.74	**0.001**
	ERT status (= Mild under-dose use)	1.04	0.744
	ERT status (= Severe under-dose use)	-6.19	**0.001**
	Any medical assistance program (= Yes)	2.01	0.483
	(Intercept)	28.24	**<0.001**
Change in SF-12 MCS	MCS at baseline	-0.32	0.105
	ERT status (= Mild under-dose use)	-0.62	0.848
	ERT status (= Severe under-dose use)	1.26	0.486
	Sex (= Female)	-2.88	0.119
	Employment/school (= Yes)	5.83	**0.002**
Change in EQ-5D index	EQ-5D index score at baseline	-0.02	0.876
	ERT status (= Mild under-dose use)	0.12	0.296
	ERT status (= Severe under-dose use)	0.11	0.245
	Sex (= Female)	-0.14	0.051
Change in EQ-5D VAS	EQ-5D VAS score at baseline	-0.69	**<0.001**
	ERT status (= Mild under-dose use)	21.72	0.105
	ERT status (= Severe under-dose use)	19.69	**0.032**
	Sex (= Female)	-22.79	**0.002**
	(Intercept)	40.64	**<0.001**

^a^ Bold values indicate statistical significance which is defined as *p*-value < 0.05.

## Discussion

### Statement of principle findings

In this study, neither mild under-dose nor severe under-dose ERT had a significant effect on the change of mental QoL. Compared with the group without ERT, mild under-dose ERT had no significant effect on the changes of physical QoL and overall health state. In contrast, severe under-dose ERT was associated with a decline of physical QoL, but an increase of overall health state in comparison with the counterpart. A higher score of physical QoL at baseline was associated with a lower physical QoL score at follow-up. The same negative association was identified in the score of overall health state. Being female was a contributor to the worsening of the overall health state, while being employed or at school was a predictor of improvement in mental QoL.

### Interpretation

We found that neither the mild under-dose group nor the severe under-dose group had a significant association with the change of mental QoL in comparison with the group without ERT. This finding was to some extent consistent with an observational study that found the mental QoL remained stable among patients with LOPD receiving ERT during the 10-year follow-up [2]. Nonetheless, the dosage of ERT was not specified in this observational study, while our contribution is that we presented detailed evidence on the dosage of ERT. We also found that the effects of mild under-dose ERT on the change of physical QoL and overall health state were not significant compared with counterpart. These findings were similar to the results of the Late-Onset Treatment Study (LOTS) trial that showed no significant difference in physical QoL between the treated and untreated group over an 18-month period [33]. However, in this randomized controlled study, the group treated with ERT had a significant improvement in 6-minute walking distance. These findings implied that improved walking distance captured in clinical settings may not reflect patients’ physical functioning in routine practice [34], or that the generic QoL instruments were insufficiently sensitive to capture changes of QoL [3]. A disease-specific instrument needs to be developed to better monitor changes of QoL among patients with LOPD on a routine basis.

By contrast, patients with severe under-dose ERT had a significant improvement in the subjective overall health state than the counterpart. This is probably because these participants had hopes and expectations toward the therapy and felt healthier after receiving ERT. This explanation is supported by the finding in patients with Spinal Muscular Disease (SMA), which is another rare neuromuscular disease [35]. SMA patients treated with nusinersen had an elevated subjective well-being at study entry triggered by hopes and expectations toward the therapy and declined during the first half year of therapy [35]. Hence, it is likely that the elevated overall health state in this study would return to the baseline level as treatment proceeds, since it represents a psychological process in association with positive life events [36]. On the other hand, severe under-dose ERT was associated with a decline in physical QoL compared with the group without ERT. Given that severe under-dose use of ERT could lead to diminished therapeutic effects [37], the decline in physical QoL probably arise from patients’ dissatisfaction or disappointment with the effect of ERT on physical functioning. Furthermore, the treatment-related adverse events, that occur in 5%-8% of treated patients with mild to moderate severity [33], could also lead to participants’ perception of exacerbated physical QoL.

We identified several factors significantly contributed to the change of QoL. Among patients with LOPD, better physical QoL and overall health state at baseline appeared to be risk factors for worse levels of corresponding QoL at follow-up. The possible explanation could be that Pompe disease is a progressive form of disease and consequently a loss of physical function at later points in life makes it harder for these patients to adapt to their situation and adjust their expectations. Female sex was a risk factor for decline in the overall health state. It is warranted to pay special attention to unmet needs in female patients with LOPD and identify its contributing socioeconomic factors. Being employed or at school was found to be a predictor of improvement in mental QoL in this study. It is probably because patients who are employed or at school have more chances to interact with other people and perform better in their social roles [8].

This study has some implications for future research, clinical practice, and policy interventions. Globally, due to variations in the study design, disease severity, and dosage of ERT, it is difficult to compare the current findings on the associations between under-dose ERT and changes of QoL with previous findings properly. More evidence is needed to focus on the dosage of ERT patients actually adopted in developing countries and assess its impact on QoL in real-world practice. Furthermore, generic QoL instruments are not sensitive enough to capture changes of QoL among adults with LOPD, and a disease-specific tool is warranted. Clinicians should closely monitor the changes of QoL among patients with under-dose ERT on a routine basis, considering the individual needs could change significantly as treatment proceeds, especially in the context of high-cost pharmacotherapy. For patients with severe under-dose ERT in China, it is necessary to assess physical functions in hospitals timely and discuss with clinicians whether to continue ERT or adjust doses. Meanwhile, except for rare disease-specific insurance and medical assistance programs supported by the local government and patient organization, more financial support is needed to enhance the reimbursement rates of ERT at the national level in China.

### Limitations

To our knowledge, this study is the first to assess the changes of QoL among adult patients with LOPD receiving under-dose ERT and identify its contributing factors.

However, this study also has some limitations. First, data on ERT use were collected using participants’ self-reported number of vials they actually used during follow-up. Although this method is simple and useful in practice, recall bias may exist when participants are filling in the survey, and thus lower the accuracy of data. Nonetheless, there is no gold standard for measuring medication adherence and each method has both advantages and disadvantages [38]. Patient self-reports of medication regimen in a questionnaire is considered the most useful method in the clinical setting [38]. Second, only a small sample size of participants was included in this study, which may limit the statistical power to detect significance and reduce the generalizability of the findings in this study. However, the inclusion of a small sample size is quite common in the area of rare disease studies. Analytical challenges include the extent to which the available sample can be viewed as representative of the entire population and whether there is statistical power to draw definitive conclusions [39]. Nonetheless, the cohort included in the current study was recruited from the only nationwide patient organization of Pompe disease in China, which is capable to reach a population of Pompe disease patients as widely as possible across the country. Future studies with a larger sample size and longer follow-up are warranted to address this limitation. Third, most of the adult patients receiving under-dose ERT included at study entry were at a relatively severe level of disease. Thus, the findings reported in this study are not generalizable to adult patients with a mild to moderate level of disease severity.

## Conclusions

A dynamic monitoring of QoL among adults with LOPD receiving under-dose ERT is necessary. Meanwhile, a Pompe-disease specific instrument should be developed based on patient experiences so that it could be sensitive enough to capture the changes of functioning and improve monitoring of QoL on a routine basis. Among patients with severe under-dose ERT, it is desirable to discuss with physicians whether to adjust treatment strategies. Customized interventions are needed to improve QoL in vulnerable subgroups, including female and unemployed people.

## Supporting information

S1 Table Demographic and socioeconomic characteristics of the study sample before propensity score matching. (DOCX)

S2 Table Classification of the level of under-dose ERT by the median ratio of vial number in the group of participants with ERT. (DOCX)
