# Supplementary material for: Association between under-dose of enzyme replacement therapy and quality of life in adults with late-onset Pompe disease in China: A retrospective matched cohort study
Source: PLoS One. 2024 Sep 17;19(9):e0310534. doi: 10.1371/journal.pone.0310534 (PMC11407662; doi:10.1371/journal.pone.0310534)
Supplement: S1 Table — (DOCX) [file pone.0310534.s001.docx]

**S1 Table. Demographic and socioeconomic characteristics of the study sample before propensity score matching**

|  | **Total** | **Not ERT** | **Under-dose ERT** | ***p-*value** |
| --- | --- | --- | --- | --- |
| **Patient number** | 70 | 59 | 11 |  |
| **Age** | 30.2(7.2) | 30.0(7.4) | 29.9(7.1) | 0.687 |
| **Sex** |  |  |  | 1.000 |
| Female | 32(45.7%) | 27(45.8%) | 5(45.5%) |  |
| Male | 38(54.3%) | 32(54.2%) | 6(54.5%) |  |
| **Reliance on devices** |  |  |  | 0.652 |
| None | 5(7.1%) | 5(8.5%) | 0(0.0%) |  |
| Some | 18(25.7%) | 16(27.1%) | 2(18.2%) |  |
| A lot | 47(67.1%) | 38(64.4%) | 9(81.8%) |  |
| **Geographic location** |  |  |  | 0.785 |
| Rural | 39(55.7%) | 32(54.2%) | 7(63.6%) |  |
| Urban | 30(42.9%) | 26(44.1%) | 4(36.4%) |  |
| Other | 1(1.4%) | 1(1.7%) | 0(0.0%) |  |
| **Wheelchair use** |  |  |  | 1.000 |
| No | 51(72.9%) | 43(72.9%) | 8(72.7%) |  |
| Yes | 19(27.1%) | 16(27.1%) | 3(27.3%) |  |
| **Ventilator use** |  |  |  | 0.675 |
| No | 12(17.1%) | 11(18.6%) | 1(9.1%) |  |
| Yes | 58(82.9%) | 48(81.4%) | 10(90.9%) |  |
| **Employment/school** |  |  |  | 0.474 |
| No | 51(72.9%) | 44(74.6%) | 7(63.6%) |  |
| Yes | 19(27.1%) | 15(25.4%) | 4(36.4%) |  |
| **Education** |  |  |  | 0.782 |
| Middle school or lower | 19(27.1%) | 17(28.8%) | 2(18.2%) |  |
| High school | 25(35.7%) | 20(33.9%) | 5(45.5%) |  |
| Above high school | 26(37.1%) | 22(37.3%) | 4(36.4%) |  |
| **Social support** |  |  |  |  |
| Tangible support | 65.9(26.3) | 65.5(27.4) | 68.2(20.6) | 0.923 |
| Emotional/informational support | 44.1(21.7) | 43.7(21.4) | 46.0(24.3) | 0.686 |
| Positive social interactions | 45.0(23.9) | 44.2(24.1) | 49.4(23.0) | 0.360 |
| Affectionate support | 46.3(26.3) | 45.6(26.4) | 50.0(26.9) | 0.570 |
| **Any rare disease-specific insurance during follow-up** |  |  |  | **0.007** |
| No | 51(72.9%) | 47(79.7%) | 4(36.4%) |  |
| Yes | 19(27.1%) | 12(20.3%) | 7(63.6%) |  |
| **Any medical assistance program during follow-up** |  |  |  | **0.010** |
| No | 63(90.0%) | 56(94.9%) | 7(63.6%) |  |
| Yes | 7(10.0%) | 3(5.1%) | 4(36.4%) |  |
| **Catastrophic health expenditure** |  |  |  | 0.100 |
| No | 30(42.9%) | 28(47.5%) | 2(18.2%) |  |
| Yes | 40(57.1%) | 31(52.5%) | 9(81.8%) |  |

Data are presented as mean (SD) for continuous variables and number (percentage) for categorical variables. Bold values indicate statistical significance which is defined as *p*-value < 0.05.
