# Supplementary material for: Association between under-dose of enzyme replacement therapy and quality of life in adults with late-onset Pompe disease in China: A retrospective matched cohort study
Source: PLoS One. 2024 Sep 17;19(9):e0310534. doi: 10.1371/journal.pone.0310534 (PMC11407662; doi:10.1371/journal.pone.0310534)
Supplement: S2 Table — (DOCX) [file pone.0310534.s002.docx]

**S2 Table. Classification of the level of under-dose ERT by the median ratio of vial number in the group of participants with ERT**

| **Patient number** | **Ratio of vial number** | **Level of under-dose ERT** |
| --- | --- | --- |
| 1 | 0.633 | Mild under-dose |
| 2 | 0.473 | Mild under-dose |
| 3 | 0.465 | Mild under-dose |
| 4 | 0.429 | Mild under-dose |
| 5 | 0.381 | Mild under-dose |
| 6 | 0.255 | Severe under-dose |
| 7 | 0.221 | Severe under-dose |
| 8 | 0.184 | Severe under-dose |
| 9 | 0.143 | Severe under-dose |
| 10 | 0.050 | Severe under-dose |
| 11 | 0.033 | Severe under-dose |
